# Reactive Nodular Fibrous Pseudotumor Mimicking Metastatic Tumor After Gastric Cancer Operation: A Case Report and Literature Review

**DOI:** 10.3389/fonc.2022.854997

**Published:** 2022-04-04

**Authors:** Junfa Chen, Yang Zhang, Lin Shi, Zhenyuan Qian, Aiping Cheng, Liping Fu

**Affiliations:** ^1^ Cancer Center, Department of Radiology, Zhejiang Provincial People’s Hospital, Affiliated People’s Hospital, Hangzhou Medical College, Hangzhou, China; ^2^ General Surgery, Cancer Center, Division of Gastrointestinal and Pancreatic Surgery, Zhejiang Provincial People’s Hospital, Affiliated People’s Hospital, Hangzhou Medical College, Hangzhou, China; ^3^ Cancer Center, Department of Nuclear Medicine, Zhejiang Provincial People’s Hospital, Affiliated People’s Hospital, Hangzhou Medical College, Hangzhou, China

**Keywords:** reactive nodular fibrous pseudotumor, gastric cancer, tomography, X-ray computed, FDG, PET/CT

## Abstract

We describe a case of reactive nodular fibrous pseudotumor (RNFP) misdiagnosed as lymph node metastasis after gastric cancer surgery. Additionally, we summarize the clinical and imaging characteristics of RNFP, combined with the literature, to improve the understanding of preoperative diagnosis. Radiological features of RNFP are a homogenous, isodense, solid mass with gradually mild enhancement on multiphasic abdominal computed tomography (CT), and slight ^18^F-FDG uptake by positron emission tomography/computed tomography (PET/CT). To the best of our knowledge, this is the first report in the English literature of a case of reactive nodular fibrous pseudotumor associated with gastric cancer and its appearance on PET/CT images.

## Introduction

Reactive nodular fibrous pseudotumor (RNFP), which is described as a rare benign tumor−like lesion, was first reported by Yantiss et al. in 2003 (1). RNFP is related to a history of abdominal surgery, injury, or inflammation, and lesions occurring after surgery for abdominal malignancy are easily misdiagnosed as tumor recurrence/metastasis. We analyzed a case of RNFP misdiagnosed as lymph node metastasis after surgery for gastric cancer and reviewed the relevant literature, with the aim of summarizing its clinical and imaging features and improving the understanding of the lesion.

## Case Presentation

In July 2018, a 54-year-old male patient with latent abdominal pain, and confirmed gastric malignant tumors by endoscopic biopsy, underwent laparoscopic radical distal gastrectomy (Billroth II gastrointestinal reconstruction) in our hospital. The postoperative pathological diagnosis was early gastric cancer with moderately to poorly differentiated adenocarcinoma in the gastric antrum, superficial bulge type (type IIa). The tumor was limited to the lamina propria.

**Figure 1:**
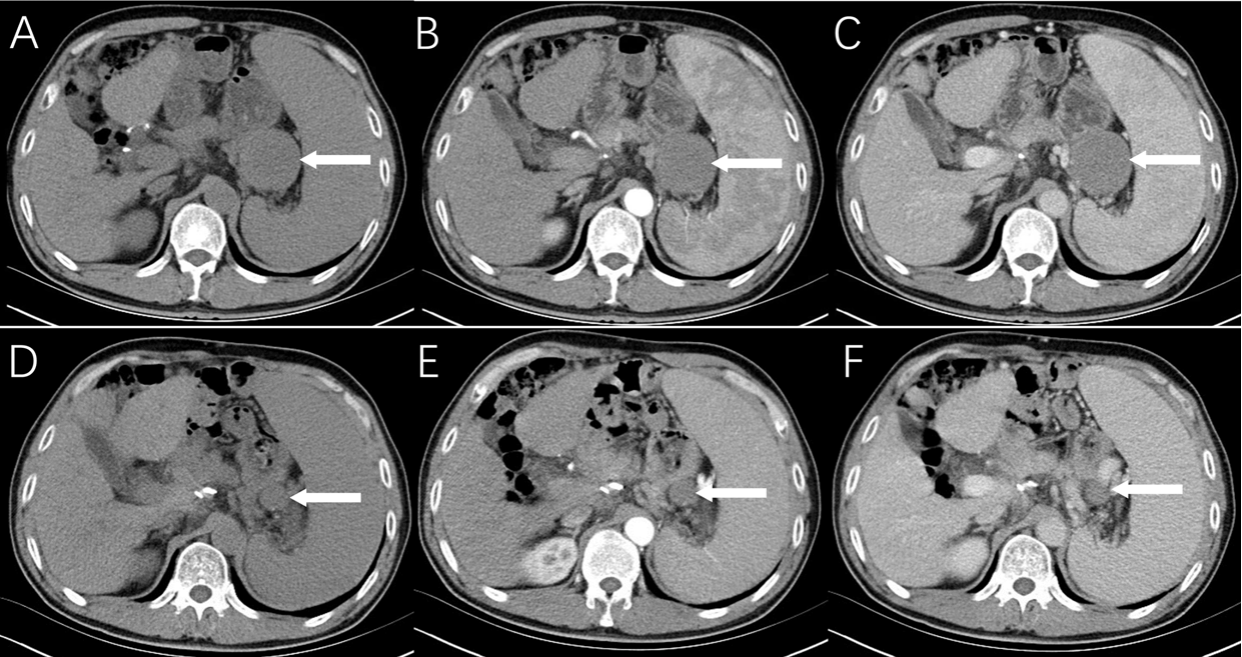
Radiological findings of reactive nodular fibrous pseudotumor. Multiphasic abdominal CT shows a solid mass measuring 5.0 cm × 5.2 cm in the operative area of gastric cancer with a relatively clear boundary, isodensity, and no necrosis **(A)**, mild enhancement in the arterial phase **(B)**, and increased contrast enhancement on portal venous phase **(C)**. On abdominal CT images **(D–F)** at 8 months ago, only a nodule with a maximum diameter of 1.0 cm was observed.

**Figure 2:**
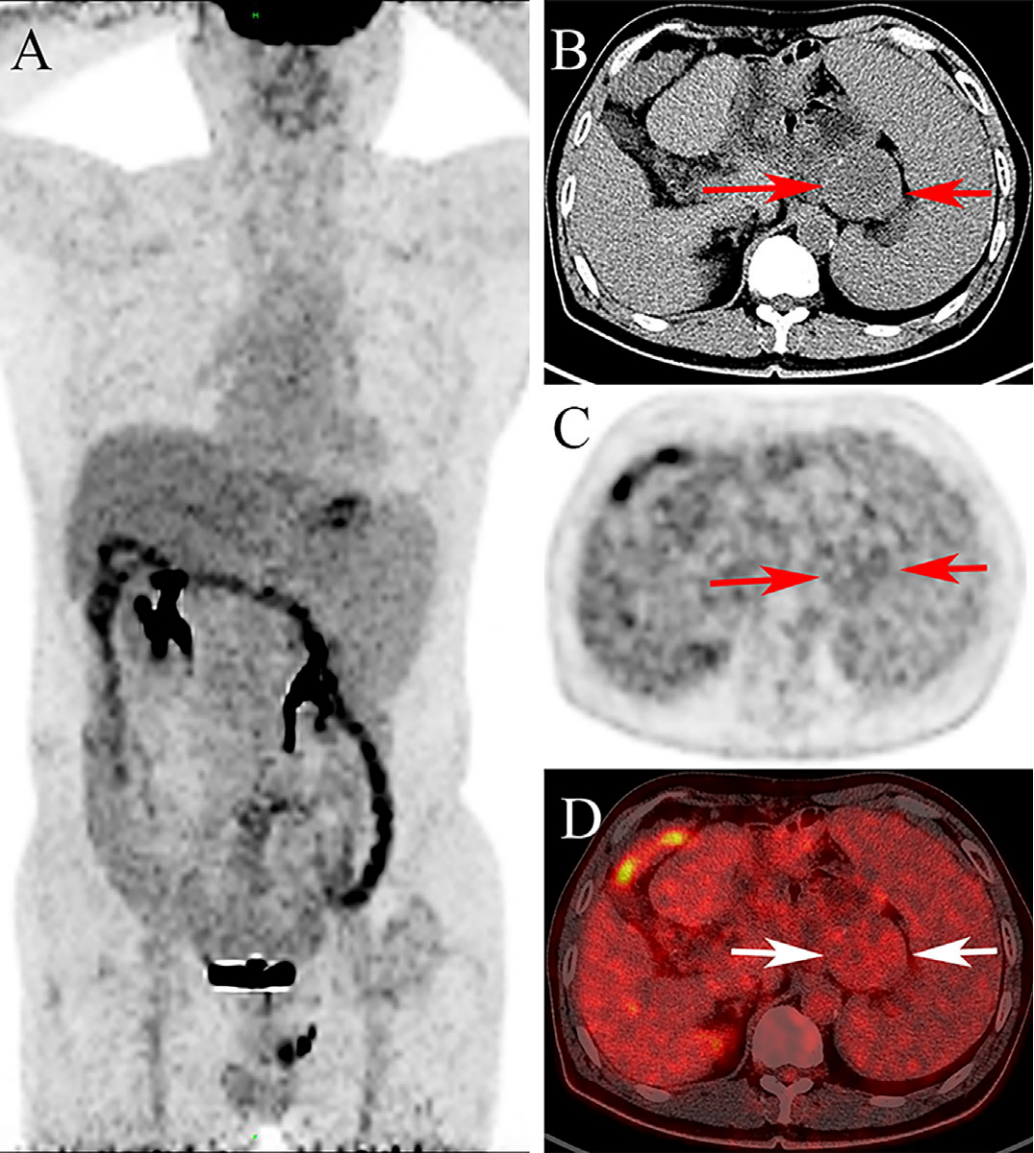
The ^18^F-FDG PET/CT images [**(A)**: maximum intensity projection image, **(B)**: the transverse CT, **(C)**: PET, **(D)**: fusion] show minimally increased FDG uptake with SUVmax of 3.6 in the lesion (arrow).

One year later, a CT scan of the abdomen revealed a round-like mass with a diameter of 5.0 cm near the anastomosis in this patient, with well-defined isodensity and no necrosis. The lesion showed slight enhancement, and the CT values of the non-contrast, arterial, and venous phases were 32HU, 43HU, and 49HU, respectively (**Figures 1A–C**). Moreover, ^18^F-FDG PET/CT (Siemens Biograph 64) was performed 60 min after intravenous injection of 310.8 MBq (8.4 mCi) of 18F-FDG. ^18^F-FDG PET/CT images revealed a minimally increased FDG uptake with SUVmax of 3.6 for a solid mass in the operative area of gastric cancer with a relatively clear boundary (**Figure 2**). Gastrofiberscopy showed residual gastritis and anastomotic stomatitis, with no sign of a tumor. Based on laboratory tests, the carcinoembryonic antigen level of the patient was slightly increased (5.1 ng/ml, normal range 0–5).

**Figure 3:**
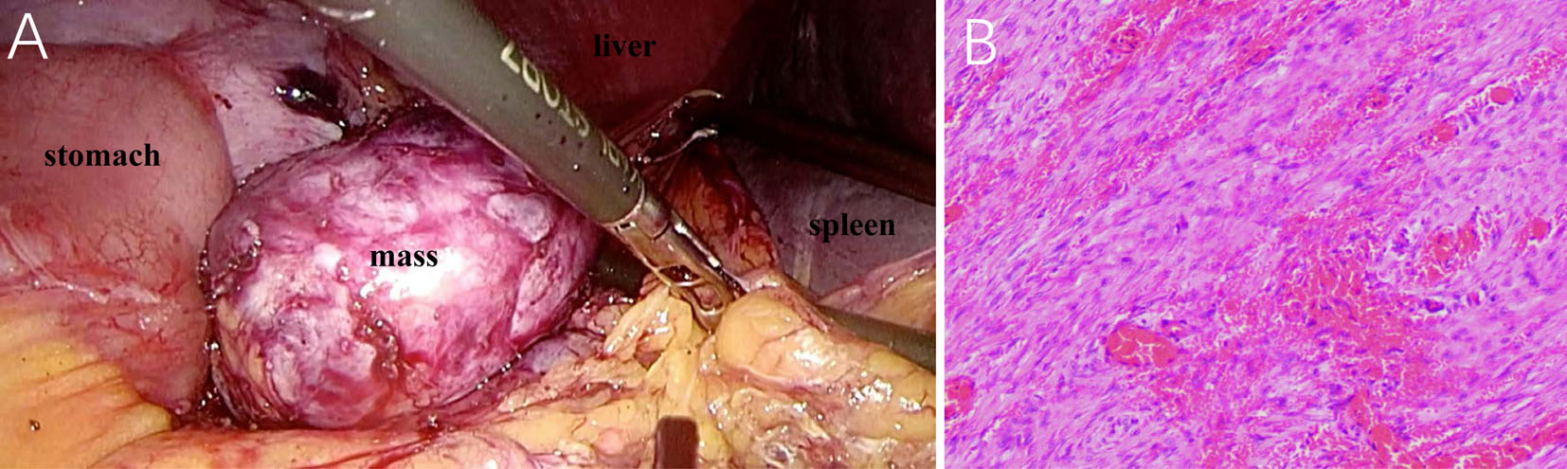
Laparoscopy presents a light-red mass with medium texture and complete capsule between the anastomotic stoma and spleen hilum **(A)**. Photomicrograph (hematoxylin–eosin stain, original magnification ×100) demonstrates the proliferation of spindle cells in the stroma of extensive collagen degeneration **(B)**. Most of the spindle cells are long fusiform, long, and deeply stained, with rare mitotic figures.

A retrospective review of previous postsurgical scans revealed a nodule with a diameter of approximately 1.0 cm (**Figures 1D–F**). Given the apparent growth of the lesion and the history of gastric cancer, lymph node metastasis was suspected, and laparoscopic splenectomy and splenic hilar lymph node dissection were performed. During the surgery, a 5.0 cm × 5.5 cm light-red mass with medium texture and clear boundaries was detected in the splenogastric space near the tail of the pancreas by laparoscopy (**Figure 3A**). The tumor was closely adhered to the spleen artery and back wall of the gastrointestinal anastomosis. Lesions on the cut section were grayish white. According to microscopic pathology, the proliferative spindle cells were disorderly arranged, with a small amount of interstitial lymphocyte infiltration. An expression of vimentin and smooth muscle actin (SMA) in most spindle cells was observed by immunohistochemical analysis, but there was no staining for S100 proteins, CD117 (c-kit), DOG1, P63, CK(Pan), or anaplastic lymphoma kinase (ALK-1 A4). The final diagnosis was reactive nodular fibrous pseudotumor (**Figure 3B**). Surgical excision was complete, and no evidence of disease was found 28 months later.

## Discussion

RNFP is considered to be a reactive benign lesion associated with previous surgical procedures or inflammatory disorders. Since 2013, twenty-seven cases of RNFP have been reported (**Table 1**) **(** 1–16), involving 16 males and 11 females, with ages ranging from 1 day to 72 years of age; the mean age was 47 years of age. Eight of these patients had a history of abdominal surgery for abdominal trauma, acute abdomen, cholecystitis, gastric stromal tumor, or colon cancer, although no lesions following a diagnosis of gastric cancer have been reported. Of the 27 cases, 12 have single lesions, and the other 15 cases involved multiple lesions. Locations were as follows: the colon or appendix (8 cases), the small bowel (7 cases), the mesentery and omentum (7 cases), the gastric wall (2 cases), and the hepatic capsule, ovary, or peripancreas (1 case each).

**Table 1:** Summary of the clinical and radiological features of RNFP reported to date.

Case #	Age (years)/sex	Number	Location	Maximum diameter (cm)	Imaging features	History	Outcome	Ref.
1	48/M	Multiple	Mesentery and jejunum	6.5	N/A	Multiple abdominal surgeries	Resection, NER	Yantiss, et al. (1)
2	50/F	Single	Peripancreatic	4.3	N/A	Multiple abdominal surgeries	Resection, NER	
3	53/M	Multiple	Mesentery and serosa of colon, ileum	5.5	N/A	Acute abdomen	Resection, NER	
4	57/M	Multiple	Mesentery and serosa of distal ileum	6.5	N/A	Acute abdomen	Incomplete resection, stable, 4 Mo	
5	71/M	Single	Mesentery of transverse colon	2.8	N/A	Abdominal surgery history	Resection, NER	
6	72/F	Multiple	Surface of small bowel and omentum	2.2	N/A	Surgery history-cholecystectomy and surgery for abdominal hernia	Resection	Zardawi, et al. (2)
7	32/M	Single	Mesentery of ascending colon	9.0	A round mass on CT	Chronic abdominal pain	Resection	Chatelain, et al. (3)
8	59/M	Single	Small bowel	3.0	N/A	Perforated and bleeding duodenal ulcer	Resection	Daum, et al. (4)
9	46/M	Single	Sigmoid colon	N/A	N/A	N/A	Resection	
10	1/M	Single	Appendix	3.0	N/A	Appendicitis	Resection	
11	68/M	Single	Cecum	10.0	N/A	N/A	Resection	
12	30/F	Multiple	Small intestine, cecum, and peritoneum	N/A	N/A	Chronic bowel obstruction with external fistula	Resection	
13	65/F	Single	Subserosa of large intestine and mesocolon	8.0	N/A	N/A	Resection	
14	22/M	Single	Ileum and omentum	7.0	N/A	N/A	Resection	
15	41/M	Single	Sigmoid colon on subserosa reaching mesocolon	6.0	N/A	N/A	Resection	
16	28/F	Multiple	Bilateral ovarian surfaces, appendix, bowel mesentery, and omentum	6.0	N/A	Chronic abdominal pain	Resection	Saglam, et al. (5)
17	60/M	Multiple	Gastric wall and lesser omentum	2.2	Isodense on unenhanced CT, with progressive contrast enhancement; moderate hypointensity on T1WI and significant hypointensity on T2WI	Incidental radiology finding	Resection; NER, 4 Mo	Gauchotte, et al. (6)
18	65/M	Single	Mesentery adjacent to the ileocecal valve	3.0	Mesenteric mass with calcifications on CT	Acute abdomen	Resection	Yin, et al. (7)
19	71/M	Multiple	Mesentery, hepatic capsule, and left paracolic gutter	6.0	N/A	Surgery history (L hemicolectomy for colon adenocarcinoma)	Resection; NER, 48 Mo	Virgilio, et al. (8)
20	54/F	Single	Hepatic hilum	4.0	N/A	Surgery history (laparoscopic cholecystectomy)	subtotal resection of the mass	Tam, et al. (9)
21	13/F	Multiple	Mesentery of the jejunum	8.8	N/A	Acute abdomen	Resection	McAteer, et al. (10)
22	45/F	Multiple	Omentum, sigmoid, and right ovary	7.0	Heterogeneous iso- to hypoattenuating masses with punctiform calcifications on CT; polylobular masses with very low signal intensity on T1WI/T2WI and hypovascular with peripheral rim-like enhancement	Intractable menometrorrhagia and abdominal pain	Resection, NER	Salihi, et al. (11)
23	16/F	Single	Gastric cardia and fundus	7.9	Solid mass with isodensity on unenhanced CT and homogenous moderate enhancement	Epigastric discomfort	Resection; NER, 24 Mo	Yi XJ, et al. (12)
24	60/F	Multiple	Mesentery, greater omentum, and serosal surface of the colon	10.0	N/A	Acute abdomen	Resection; NER, 24 Mo	Yan, et al. (13)
25	71/M	Single	Between the diaphragm, transverse colon and stomach	19.5	N/A	Abdominal surgery history	Resection; NER, 8 Mo	Ciftci, et al. (14)
26	65/F	Single	Gastrohepatic ligament	1.6	N/A	Surgery history (wedge resection of the stomach)	Resection	Moodley, et al. (15)
27	17/M	Multiple	Transverse mesocolon and peritoneum	10.0	Inhomogeneous calcified mass with rosary bead enhancement on CT	Acute abdomen	Resection; NER, 60 Mo	Girsowicz, et al. (16)

M, male; F, female; Mo, months; N/A, not available; NER, no evidence of recurrence.

Microscopic examination of RNFP reveals proliferation of stellate or spindle cells with a keloid-like appearance in a dense collagenous background, accompanied by infiltration of lymphocytes and plasma cells. Immunohistochemistry shows positive staining for vimentin and smooth muscle actin and negative staining for CD34 and S-100. The immunohistochemical characteristics of the present case are consistent with previous results (10).

Of the cases reported thus far, only 4 had CT and/or MR images. Combined with our case and the literature (6, 11, 12, 16), the imaging features are as follows. On plain CT, the lesion is isodense, with progressively mild enhancement observed on postcontrast CT. MR manifestations are related to the content of fibroblasts in the RNFP. In general, the lesion shows homogenous hypointensity on T1WI and a mixed high-signal shadow with a slightly hypointensity area on T2WI. Enhancement mode results are the same as those by CT. If the lesion is rich in fibroblastic components, the appearance of a markedly low signal on T2WI has certain characteristics. There is no PET/CT description of RNFP in the literature. In the present case, PET/CT images showed a round-like mass with slightly increased FDG metabolism. The features of isodensity and progressively mild enhancement on CT images corresponded with the literature.

RNFP should be differentiated from lymph node metastasis related to gastric cancer and extragastrointestinal stromal tumors (EGIST). Enlarged lymph nodes are irregular in shape, with marginal lobulation or spiny processes. Uneven high enhancement with central necrosis and ring enhancement when the diameter of the node is more than 10 mm are typical; PET/CT FDG metabolism is significantly increased. Another neoplastic entity to consider in differential diagnosis is an EGIST, which is commonly a unique large mass with a round or irregular shape and uneven density. Cystoid degeneration and necrosis are common. EGIST exhibits significant enhancement during the arterial phase due to the hypervasculature present, in contrast to the mild enhancement of RNFP.

Surgical resection is currently an effective treatment for RNFP. No cases of RNFP recurrence or metastasis have been reported to date.

## Conclusion

RNFP is rare and may be related to postoperative fibroinflammation. We report for the first time a reactive nodular fibrous pseudotumor associated with surgery for gastric cancer and its appearance on PET/CT images. Awareness of the imaging features of RNFP is important for its diagnosis. For patients who have been treated with surgery for abdominal malignant tumor, to avoid misdiagnosis and overtreatment, the possibility of RNFP should be considered if the mass is found to have the above imaging findings.

## Data Availability Statement

The raw data supporting the conclusions of this article will be made available by the authors, without undue reservation.

## Ethics Statement

The studies involving human participants were reviewed and approved by the Zhejiang Provincial People’s Hospital, Affiliated People’s Hospital, Hangzhou Medical College. The patients/participants provided their written informed consent to participate in this study.

## Author Contributions

YZ and LS performed the image acquisition. ZQ collected the clinical and pathological data. JC and LF performed the image analysis and wrote the manuscript. AC and LF revised the final manuscript. All authors contributed to the article and approved the submitted version.

## Funding

This research was supported by the Zhejiang Provincial Natural Science Foundation of China (Nos. LGC22H180002, GC20H180003 and Y21H160246) and Zhejiang Medical and Health Science and Technology Project (Nos. 2020KY406, 2021KY508).

## Conflict of Interest

The authors declare that the research was conducted in the absence of any commercial or financial relationships that could be construed as a potential conflict of interest.

## Publisher’s Note

All claims expressed in this article are solely those of the authors and do not necessarily represent those of their affiliated organizations, or those of the publisher, the editors and the reviewers. Any product that may be evaluated in this article, or claim that may be made by its manufacturer, is not guaranteed or endorsed by the publisher.
